# Investigation into Early Steps of Actin Recognition by the Intrinsically Disordered N-WASP Domain V

**DOI:** 10.3390/ijms20184493

**Published:** 2019-09-11

**Authors:** Maud Chan-Yao-Chong, Dominique Durand, Tâp Ha-Duong

**Affiliations:** 1 BioCIS, University Paris-Sud, CNRS UMR 8076, University Paris-Saclay, 92290 Châtenay-Malabry, France; maud.chan-yao-chong@u-psud.fr; 2 Institute for Integrative Biology of the Cell (I2BC), CEA, CNRS, University Paris-Sud, University Paris-Saclay, 91190 Gif-sur-Yvette, France; dominique.durand@i2bc.paris-saclay.fr

**Keywords:** intrinsically disordered protein, protein–protein interaction, molecular docking, molecular dynamics

## Abstract

Cellular regulation or signaling processes are mediated by many proteins which often have one or several intrinsically disordered regions (IDRs). These IDRs generally serve as binders to different proteins with high specificity. In many cases, IDRs undergo a disorder-to-order transition upon binding, following a mechanism between two possible pathways, the induced fit or the conformational selection. Since these mechanisms contribute differently to the kinetics of IDR associations, it is important to investigate them in order to gain insight into the physical factors that determine the biomolecular recognition process. The verprolin homology domain (V) of the Neural Wiskott–Aldrich Syndrome Protein (N-WASP), involved in the regulation of actin polymerization, is a typical example of IDR. It is composed of two WH2 motifs, each being able to bind one actin molecule. In this study, we investigated the early steps of the recognition process of actin by the WH2 motifs of N-WASP domain V. Using docking calculations and molecular dynamics simulations, our study shows that actin is first recognized by the N-WASP domain V regions which have the highest propensity to form transient α-helices. The WH2 motif consensus sequences “LKKV” subsequently bind to actin through large conformational changes of the disordered domain V.

## 1. Introduction

Intrinsically disordered proteins (IDPs) play important roles in the regulation of many biological processes, such as cell growth, cell signaling, and cell survival. To exert these functions, their intrinsically disordered regions (IDRs) often bind to different proteins with high specificity and low affinity [1,2,3,4]. In many cases, it is observed that IDRs adopt well structured conformations when bound to their partners [5]. Segments that undergo such a disorder-to-order transition upon binding are frequently called Molecular Recognition Features (MoRFs) in the literature [4,6,7,8,9,10].

A typical IDR with a MoRF is the WASP-homology 2 (WH2) motif, which is found in about 50 proteins [11]. WH2 motifs are actin-binding modules of about 30–50 residues that are key players in regulation of the cytoskeleton actin polymerization, dynamics, and organization [11,12,13]. Proteins of the WH2 family can contain one to four WH2 motifs, each being able to bind one G-actin monomer (Table S1). In unbound state, WH2 motifs are intrinsically disordered, and, in complex with actin, they all share a similar binding mode: their N-terminal part folds into an α-helix which interacts with the barbed face of actin, between subdomains 1 and 3, while their central consensus sequence “LKKV” has an extended conformation which lies on the actin’s surface, between subdomains 1 and 2 [11,14,15] (see Figure 2B). Although these actin–WH2 motif structures were determined by X-ray diffraction, the common folding of different WH2 motifs upon binding to actin indicates, with reasonable confidence, that it is probably similar to the one adopted in solution.

It should be noted that, when a WH2 motif or a peptide construct encompassing a WH2 motif is co-crystallized with actin, only the coordinates of about 20 residues, generally from the beginning of the helical segment to the consensus sequence “LKKV”, were resolved in most crystallographic complexes (Table S1). Only the crystallographic structures 2A41, 2D1K, and 5YPU contain almost all residue coordinates of the co-crystallized WH2 motifs. The absence in most crystallographic structures of atomic coordinates for regions after the consensus sequence “LKKV” indicates that they probably keep a highly flexible and disordered conformation upon binding to actin, forming so-called fuzzy complexes. Questions that could be raised here are: What is the conformational dynamics of these invisible regions? Are they interacting with actin, and, if so, with which residues?

A more general and still debated question regarding IDRs concerns the mechanism of their specific binding to their partners. The formation of IDP–protein complexes can indeed follow a pathway between two possible mechanisms [16]: the “induced fit” pathway, in which the disordered region binds to its partner and folds into an ordered structure on its surface, and the “conformational selection” mechanism, in which the folded structure preexists among the ensemble of conformations of the unbound IDP and is recognized by the protein partner. However, the observation of preexisting structured segments in IDRs does not necessarily prove that the binding proceeds by a direct conformational selection [17]. For example, an alternative mechanism could be that the protein partner first binds to any IDR region and slides to the specific binding site which has the correct complementary conformation [18]. Thus, closer investigations are required to gain insight into the early events and pathways of the IDP–protein recognition mechanism.

In this report, we address these issues in the case of the verprolin homology domain (V) of the Neural Wiskott–Aldrich Syndrome Protein (N-WASP), which has two WH2 motifs. With the Arp2/3 complex, N-WASP stimulates actin filament branching and the formation of dendritic networks of filaments that shape or deform cell membranes in several cellular processes, such as cell motility or endocytosis [19,20]. The 505-residue sequence of the human N-WASP can be decomposed into seven domains: a primary WASP homology domain WH1 (segment 1–150), a basic domain B (186–200), a GTPase-binding domain GBD (203–274), a proline-rich domain PRD (277–392), a verprolin homology domain V (405–450), a cofilin homology domain C (451–485), and an acidic domain A (486–505) [21,22]. N-WASP domain V binds and recruits G-actin monomers, while domains CA are attached to the Arp2/3 complex. These associations allow the nucleation of new branch filaments [19,23,24]. N-WASP domain V is composed of two WH2 motifs (Table S1), each being able to bind one G-actin [25,26,27]. Interestingly, the presence of two WH2 motifs in N-WASP domain V induces more rapid actin polymerization than the other proteins of the WASP family which have only one WH2 motif [28]. However, the structural mechanism by which a tandem of WH2 motifs binds two actin monomers and accelerates polymerization and branching is not completely elucidated.

Two crystallographic structures of the N-WASP WH2 tandem in complex with actin are available in the Protein Data Bank: a 1:1 actin–domain VC (2VCP [27]) and a 2:1 actin–WH2 tandem (3M3N [26]). Nevertheless, in both 2VCP and 3M3N structures, we emphasize again that only about 20 residues of each WH2 motif, from the helical N-terminal part to the consensus sequence “LKKV”, could be resolved by X-ray experiments (Table S1). It should be noted that the actin dimer in 3M3N complex has an overall longitudinal arrangement similar to that one in actin filament [26]. This suggests that N-WASP domain V might favor the formation of actin dimers in a longitudinal filament-like conformation, which might accelerate actin polymerization. However, to confirm this scenario, a detailed description of the formation of the 1:1 and 2:1 actin–domain V complexes in solution is required.

Previously, we structurally characterized the unbound state of a construct encompassing N-WASP domain V (Figure S1) by combining various biophysical techniques [29]. Multiple molecular dynamics (MD) simulations allowed generating a conformational ensemble of this construct (which we continue to call “N-WASP domain V” for simplicity) in very good agreement with both NMR chemical shifts and SAXS intensity measurements. In this ensemble, several conformations were identified with transient α-helices in the WH2 motifs, suggesting that these secondary structures might be selected by actin during the recognition process. We query here the validity of this hypothesis and, more generally, investigate the early events of actin recognition by these α-MoRFs, using protein–protein docking calculations and multiple MD simulations. In addition, since N-WASP has a tandem of WH2 motifs, we examine the possible molecular pathways leading to the ternary complex of domain V with two actins.

## 2. Results

NMR experiments and MD simulations previously showed that unbound N-WASP domain V has two transient α-helical structures (one per WH2 domain) at regions 10–15 and 37–43 corresponding to residues 407–412 and 434–440 in the whole protein sequence (Figure S1) [29].

### 2.1. Monomeric Actin–Domain V Encounter Complexes Generated by Docking Calculations

To examine whether these two helical MoRFs are preferential recognition sites for actin, we blindly docked the 527 most populated clusters of N-WASP domain V conformational ensemble (derived from MD simulations with the A03ws force field [29]) onto the actin chain B extracted from the PDB structure 2VCP [27]. Each docking generated about 1300 different poses of domain V on actin, yielding a total number of 702,920 encounter complexes. The likeliness of these complexes was evaluated with the scoring function 2/3B${}^{best}$ InterEvScore [30]. We delineated the 1% of complexes (i.e., 7030 conformers) having the highest 2/3B${}^{best}$ score as the most probable actin–domain V structures. It could be noted that, when compared to the 527 cluster representative structures, the domain V conformations that are retrieved in the 7030 most probable complexes are sightly more compact, as indicated by the radius of gyration distributions (Figure S2), indicating that extended conformations of domain V did not particularly favor their binding to actin. At the local level, the difference in probability for residues to be in α-helix, between the two ensembles of 527 clusters and of 7030 ligands, appears quite small and may not be significant (Figure S2).

**Figure 1:**
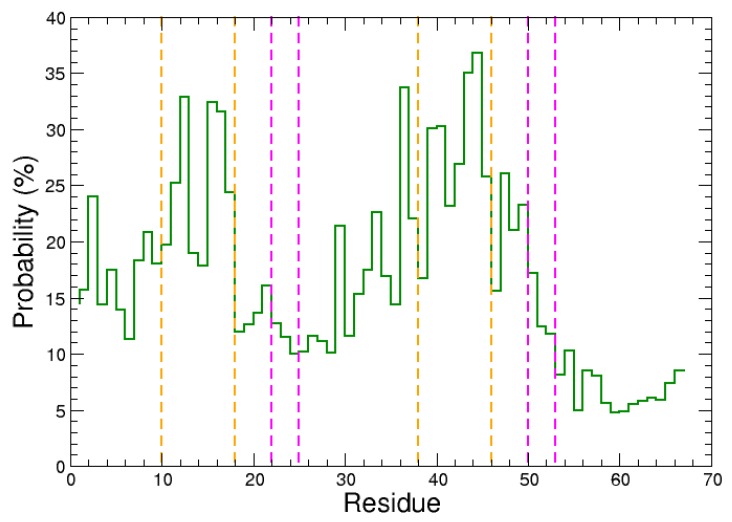
Probability of the N-WASP domain V residues to be distant by less than 4 Å from actin. Orange and magenta dashed lines indicate the protein regions in α-helix (as revealed by the X-ray structure 2VCP [27]) and the consensus sequences “LKKV” [14,31], respectively.

We first analyzed the residues at the protein–protein interface in the 7030 most probable complex structures. The probability of N-WASP domain V residues to be in contact with actin was computed, as plotted in Figure 1. Clearly, it can be observed that actin preferentially recognizes two regions of domain V which can be delimited by residues 8–18 and 37–50. The first binding site is shorter than the second one, which might be related to the difference in propensity of the two WH2 motifs to form α-helical structures (Figure S2). Nevertheless, when the two regions with high probability to be contacted by actin are compared, a consensus sequence can be identified as the most probable recognition site for actin: ${}^{9}$KAALLDQIRE${}^{18}$ and ${}^{37}$RDALLDQIRQ${}^{46}$ in the first and second WH2 motif, respectively. It is worth noting that both recognition segments exhibit a similar pattern in which a positively charged residue (K9 or R37) precedes two moderate probability residues (A10/A11 or D38/A39), followed by two high probability hydrophobic residues (L12/L13 or L40/L41) and again two moderate probability ones (D14/Q15 or D42/Q43), before two other high probability residues (I16/R17 or I44/R45). This pattern suggests that the domain V recognized regions are rather α-helical structures than short linear motifs (SLiMs) in coil or extended conformations. The chemical nature of the mentioned residues also indicates that the central parts of the recognized segments are amphiphilic helices with their hydrophobic faces in contact with actin.

Besides, it could be noted that, among the most probable complexes, the conserved residues ${}^{22}$LKKV${}^{25}$ and ${}^{50}$LKSV${}^{53}$ have significantly lower probability to be in contact with actin than the two previous binding sites (Figure 1). This suggests that, after the recognition of regions 9–18 or 37–46 by actin, the N-WASP consensus sequences “LKKV” should move and anchor to the actin’s surface in a second step. This scenario was further examined using MD simulations, as presented in the next section.

**Figure 2:**
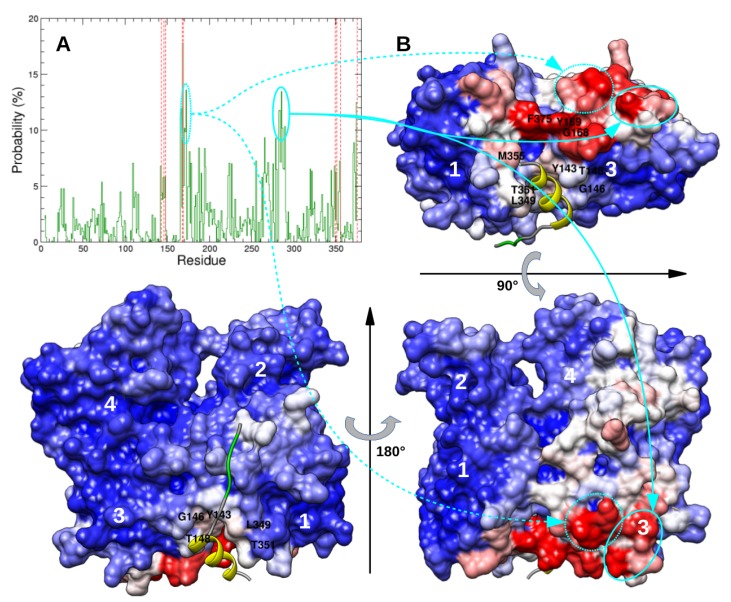
(**A**) Probability of actin residues to be distant by less than 4 Å from domain V regions 9–18 or 37–46. Red dashed lines indicate actin residues in contact with N-WASP helical segment in structure 2VCP [27]. (**B**) Views of actin’s surface colored proportionally to previous probabilities. Blue, white, and red colors indicate actin residues with low, intermediate, and high probabilities to be contacted by domain V, respectively. As a reference, yellow and green ribbons represent the second WH2 motif helical region and conserved sequence LKSV as observed in 2VCP [27].

Before that, we investigated the preferential location of the two N-WASP regions 9–18 and 37–46 on actin’s surface. To that end, the probability that actin residues are contacted by one of these two segments was computed over the 7030 most probable complexes predicted by docking, as plotted in Figure 2A. Among the actin residues which are frequently contacted by regions 9–18 and 37–46, we retrieved those (Y143, G146, T148, G168, Y169, L349, T351, M355, and F375) which make contacts with the N-WASP segment 37–46 in structure 2VCP [27]. However, we also observed that segments 9–18 or 37–46 can bind to other patches of the actin’s surface with high probability, notably residues 171–173 and 283–290, which are not close to the cognate binding site (Figure 2). These observations could arise from various factors, including limitations of the rigid-body docking procedure and imperfections of the coarse-grained scoring function. This could be also related to the fact that, in most selected conformations of N-WASP domain V used in docking calculations, segments 9–18 and 37–46 were not fully helical, unlike in the crystallographic complex (Figure S2). This might favor the binding to pockets of the actin’s surface with no particular shape, to the detriment of the groove that is expected to accommodate the WH2 motif helices. In these cases, the conformational transition of these N-WASP regions toward full α-helices might not lead to stable complexes. Besides, it could be noted that these non-specific binding sites on actin monomer also extend over the actin–actin interface in longitudinal dimers and, therefore, might be less observed in such actin assemblies.

Overall, docking calculations of representative conformations of free domain V on actin monomer yielded many encounter complexes in which N-WASP segments 9–18 and 37–46 are preferentially bound to actin, but to both specific and non-specific sites. In these encounter complexes, consensus sequences “LKKV” have low probability to be in contact with actin, whereas they are found attached to actin in all available crystallographic complex structures. This suggests a two-step association mechanism involving large conformational rearrangements of domain V after the formation of a productive encounter complex with either segment 9–18 or 37–46 in cognate binding site of actin.

### 2.2. Identification and MD Simulations of Productive Actin–Domain V Encounter Complexes

The binding mechanism of N-WASP domain V to actin was further investigated using MD simulations of productive encounter complexes selected on the basis of the position and orientation of regions 9–18 or 37–46 in the cognate actin binding groove. More specifically, among the 7030 most probable complexes generated by docking, we identified those with residues 9–18 or 37–46 contacting at least six actin residues over the nine observed in contact with the N-WASP region 37–46 in the X-ray structure (Y143, G146, T148, G168, Y169, L349, T351, M355, and F375). We found a total of 194 complexes which have one of the two recognized segments in contact with at least six of the nine actin hot-spot residues. However, in a large number of these complexes, the segment 9–18 or 37–46 is oriented in the opposite direction of the crystallographic helix, so that the consensus sequence “LKKV” would not be able to reach its cognate binding site. Thus, we further filtered the 194 complexes based on the angle between the principal axis of segment 9–18 or 37–46 and that one of the helical region 37–46 in crystal. We obtained 16 and 18 complexes in which this angle is lower than 30${}^{\circ }$ for N-WASP regions 9–18 and 37–46, respectively (Tables S2 and S3).

**Figure 3:**
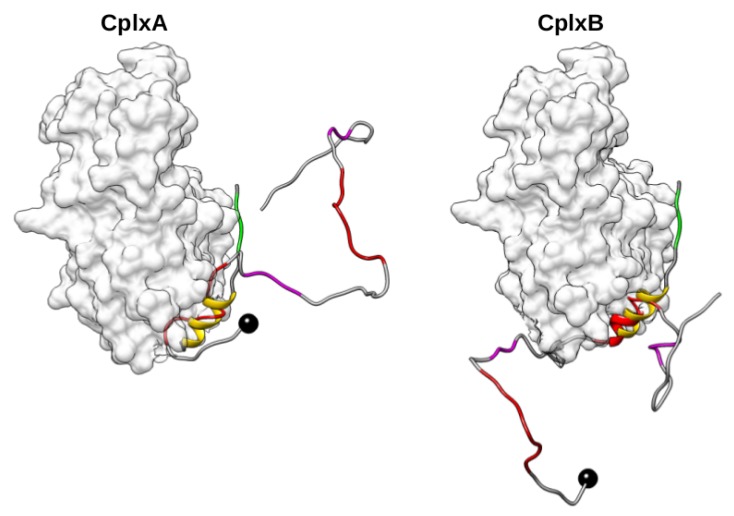
Side view of the two best 1:1 actin–domain V encounter complexes with N-WASP segment 9–18 (**left**) or 37–46 (**right**) located and oriented as in structure 2VCP. Black balls are N-terminal Cα-atoms of domain V. Red and magenta ribbons represent its regions 9–18 or 37–46 and consensus sequences “LKKV”, respectively. As a reference, yellow and green ribbons indicate the helical and ${}^{50}$LKSV${}^{53}$ regions of domain VC in 2VCP.

In these 34 productive actin–domain V encounter complexes, the recognized regions 9–18 and 37–46 are surprisingly not completely folded in α-helix, but can have various local conformations with 0–6 over 10 residues in helical structures. Nevertheless, it should be noted that the lack of helical residues is often balanced by several residues with a turn motif. This is notably the case for four over the five complexes which have region 9–18 or 37–46 RMSD lower than 5 Å relative to the crystallographic structure (Tables S2 and S3). In the 34 actin–domain V complexes, the consensus segments “LKKV” are variously far off from their cognate binding site on actin, as indicated by their RMSD values ranging from 8.7 to 37.7 Å. To study the complete association process of N-WASP WH2 motifs, we performed MD simulations of actin–bound domain V conformational changes starting from the two structures which have region 9–18 or 37–46 with the lowest RMSD relative the structure 2VCP (Figure 3). These selected productive encounter complexes are hereafter denoted CplxA and CplxB.

**Figure 4:**
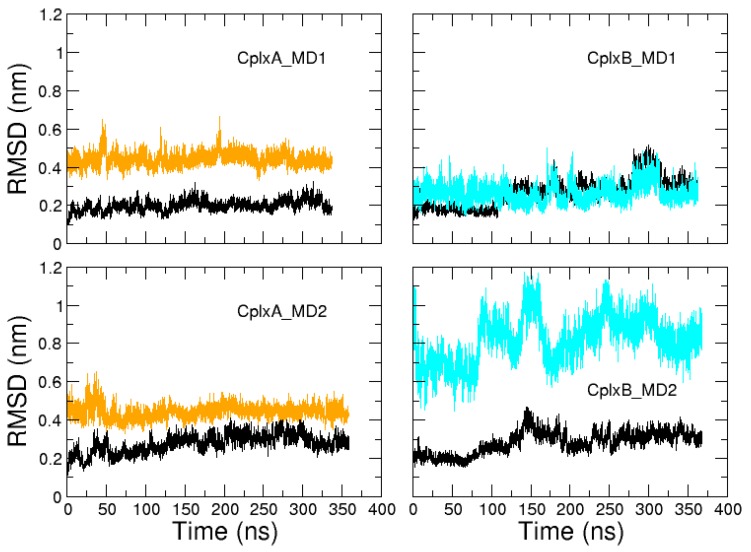
Time evolutions of RMSD relative to structure 2VCP, after fitting MD trajectories on crystallographic actin, for actin (black) and segments 9–18 (orange) and 37–46 (cyan) of N-WASP domain V.

For each selected encounter complex, two MD simulations of about 350 ns were performed from the same coordinates but with different initial velocities. These four simulations will be referred to as CplxA_MD1, CplxA_MD2, CplxB_MD1, and CplxB_MD2. In all complex trajectories, the actin tertiary structure remains stable, with RSMD relative to structure 2VCP fluctuating below 5.2 Å (Figure 4). Regarding the N-WASP regions 9–18 and 37–46 (which are bound to actin in CplxA and CplxB, respectively), their position and orientation are maintained in the actin binding site in three over four simulations (CplxA_MD1, CplxA_MD2, and CplxB_MD1), as indicated by their average RMSD values relative to the complex 2VCP (4.4, 4.4, and 2.7 Å, respectively). A visual inspection of the CplxB_MD2 trajectory showed that segment 37–46 slid toward the bottom of actin, explaining its higher RMSD (8.2 Å on average). For the three other simulations, the N-WASP regions 9–18 and 37–46 remain attached to their binding site after the formation of productive encounter complexes.

**Figure 5:**
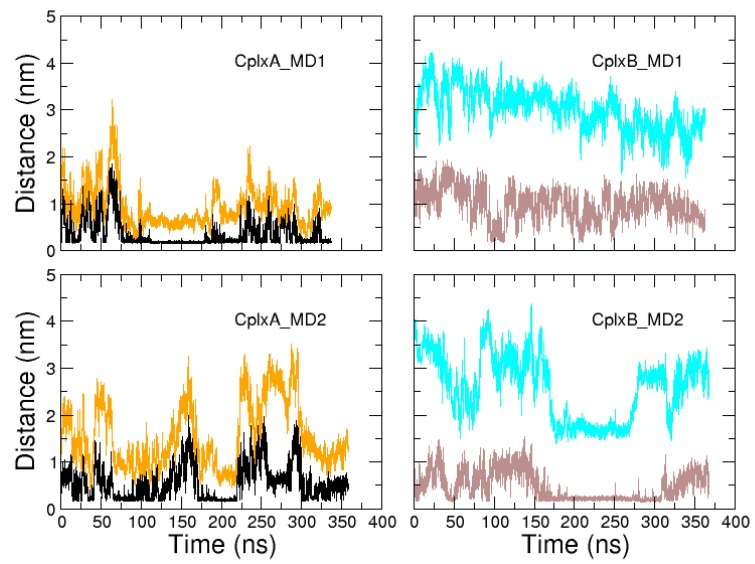
Time evolutions of minimal distance to actin of segments ${}^{22}$LKKV${}^{25}$ (black) and ${}^{50}$LKSV${}^{53}$ (brown) of N-WASP domain V. RMSD relative to structure 2VCP, after fitting trajectories on actin, are also displayed as a function of time for segments ${}^{22}$LKKV${}^{25}$ (orange) and ${}^{50}$LKSV${}^{53}$ (cyan).

Next, we monitored the dynamics of residues ${}^{22}$LKKV${}^{25}$ and ${}^{50}$LKSV${}^{53}$ relative to their cognate binding site on actin. As shown in Figure 5, segments ${}^{22}$LKKV${}^{25}$ and ${}^{50}$LKSV${}^{53}$ had large amplitude motions in all four simulations, without reaching stable bound positions on actin. Strikingly, the minimal distance to actin of these residues and their RMSD relative to structure 2VCP seem to be highly correlated, which can be explained as follows: Once N-WASP domain V helical region 9–18 or 37–46 is correctly positioned and oriented in its cognate binding site, if segment ${}^{22}$LKKV${}^{25}$ or ${}^{50}$LKSV${}^{53}$ is detached from actin’s surface, it is largely free to move in solvent, accounting for large RMSD values. However, when it is bound to actin, its accessible space is narrowed down to a region close to the cognate site on actin, decreasing the RMSD relative to X-ray structure. However, in none of simulations, these segments were observed to persistently bind to their cognate binding site: In simulations CplxB_MD1 and CplxB_MD2, RMSD of residues ${}^{50}$LKSV${}^{53}$ relative to the crystallographic structure never decreased below 13.8 Å. The observed large RMSD values are mainly due to the fact that segment ${}^{50}$LKSV${}^{53}$ is, most of the time, detached from actin’s surface in simulations of CplxB. In simulations of CplxA, segment ${}^{22}$LKKV${}^{25}$ was able to reach its cognate site, with minimal RMSD of 2.4 and 4.3 Å in CplxA_MD1 and CplxA_MD2, respectively, but these associations were only transient (Figure 5). Overall, in three over four simulations, residues ${}^{22}$LKKV${}^{25}$ or ${}^{50}$LKSV${}^{53}$ were observed to bind the actin’s surface during quite long periods, but not necessarily at their cognate locations, confirming that these N-WASP segments are not primary recognition sites for actin. Finally, we should point out that the auto-correlation functions of minimal distances to actin of residues ${}^{22}$LKKV${}^{25}$ or ${}^{50}$LKSV${}^{53}$ are characterized by relaxation times of 102, 126, 164, and 133 ns for simulations CplxA_MD1, CplxA_MD2, CplxB_MD1, and CplxB_MD2, respectively. This notably indicates that the two short simulations of CplxA still provide reliable information about the dynamics of segment ${}^{22}$LKKV${}^{25}$.

**Figure 6:**
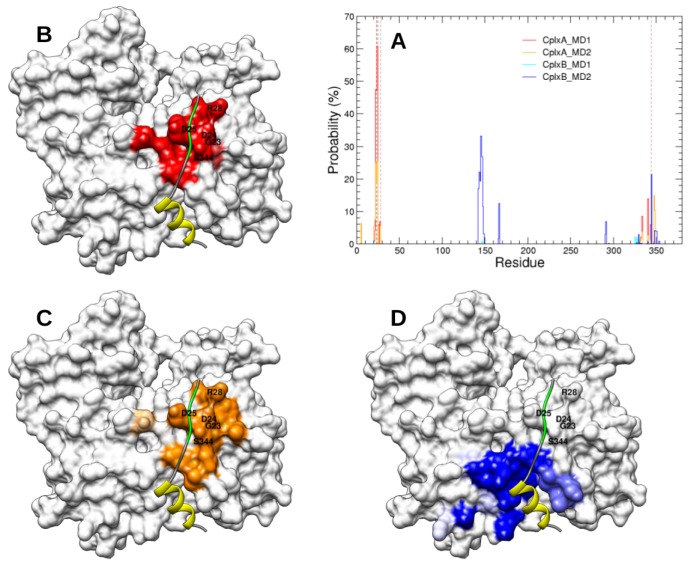
(**A**) Probability of actin residues to be distant by less than 4 Å from N-WASP segments ${}^{22}$LKKV${}^{25}$ or ${}^{50}$LKSV${}^{53}$ in CplxA_MD1 (red), CplxA_MD2 (orange), CplxB_MD1 (cyan), and CplxB_MD2 (blue). Brown dashed lines indicate the actin residues (G23, D24, D25, R28, and S344) in contact with N-WASP segment ${}^{50}$LKSV${}^{53}$ in structure 2VCP [27]). (**B**–**D**) Front views of the actin’s surface colored proportionally to the previous probabilities. Red, orange, and blue colors indicate actin residues with high probabilities to be contacted by N-WASP segments ${}^{22}$LKKV${}^{25}$ or ${}^{50}$LKSV${}^{53}$ in simulations CplxA_MD1 (**B**), CplxA_MD2 (**C**), and CplxB_MD2 (**D**), respectively. As a reference, yellow and green ribbons represent the helical region and the conserved sequence LKSV of the second WH2 motif observed in structure 2VCP [27].

The actin residues that have high probabilities to be contacted by these segments are shown in Figure 6. In both simulations of CplxA, segment ${}^{22}$LKKV${}^{25}$ was found in contact with several actin residues close to the cognate binding site. In contrast, due to the sliding of region 37–46 toward the bottom of actin in simulation CplxB_MD2, the segment ${}^{50}$LKSV${}^{53}$ is too far to reach and bind its cognate site on actin. All together, despite their limited number and length, our simulations suggest that CplxA (which has the N-WASP helical region 9–18 recognized by actin) is likely a productive encounter complex that can lead to a subsequent binding of segment ${}^{22}$LKKV${}^{25}$ to its specific site on actin. In contrast, simulations of CplxB suggest that the complete binding of N-WASP second WH2 motif is less favorable than for the first WH2 motif. Beyond the limited statistics, this could result from the fact that segment ${}^{50}$LKSV${}^{53}$ is less positively charged than ${}^{22}$LKKV${}^{25}$, whereas their cognate binding site on actin has two negatively charged residues (D24 and D25). Another possible explanation is that N-WASP region 37–46 has a higher propensity to form α-helices than segment 9–18. This would increase the stiffness of the second WH2 motif that might restrict the motion of residues ${}^{50}$LKSV${}^{53}$ and their ability to reach their cognate binding site on actin.

**Figure 7:**
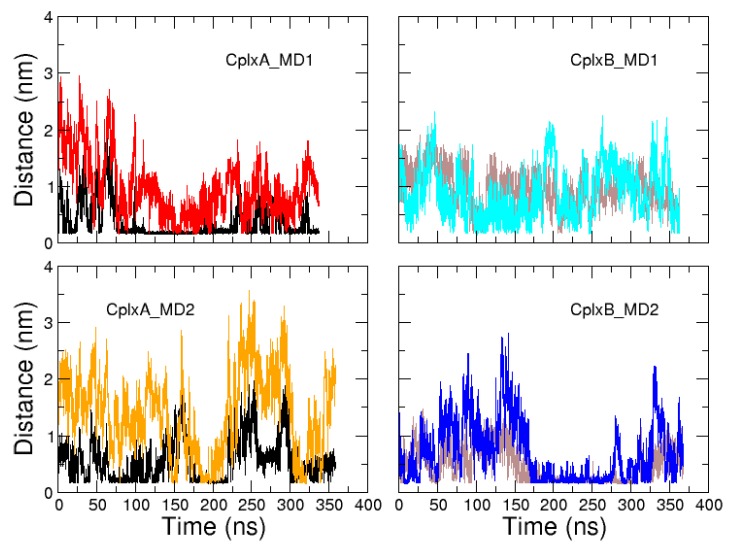
Time evolutions of minimal distances between actin and segment ${}^{28}$NSRPVS${}^{33}$ in simulations of CplxA (red and orange lines) and segment ${}^{56}$GQESTP${}^{61}$ in simulations of CplxB (cyan and blue lines). For comparison, time evolutions of minimal distances between actin and segments ${}^{22}$LKKV${}^{25}$ and ${}^{50}$LKSV${}^{53}$ are displayed with black and brown lines, respectively.

Finally, we studied the dynamics of domain V regions ${}^{28}$NSRPVS${}^{33}$ and ${}^{56}$GQESTP${}^{61}$ following the conserved sequences ${}^{22}$LKKV${}^{25}$ and ${}^{50}$LKSV${}^{53}$, respectively. Indeed, as mentioned in the introduction, most crystallographic structures of actin–WH2 motif lack atomic coordinates for regions after the consensus sequence “LKKV”, indicating that they are highly flexible in their bound state. We thus characterized the preferential location of these two regions on actin’s surface in our MD simulations. Figure 7 plots the minimal distance of regions ${}^{28}$NSRPVS${}^{33}$ and ${}^{56}$GQESTP${}^{61}$ to actin as a function of time in CplxA and CplxB simulations, respectively. It can be observed that these two regions mostly contact the actin’s surface when the preceding conserved sequences ${}^{22}$LKKV${}^{25}$ or ${}^{50}$LKSV${}^{53}$ are already attached to actin, except in CplxB_MD1. In the latter, residues ${}^{56}$GQESTP${}^{61}$ make frequent contacts with actin when segment ${}^{50}$LKSV${}^{53}$ is not bound to actin.

**Figure 8:**
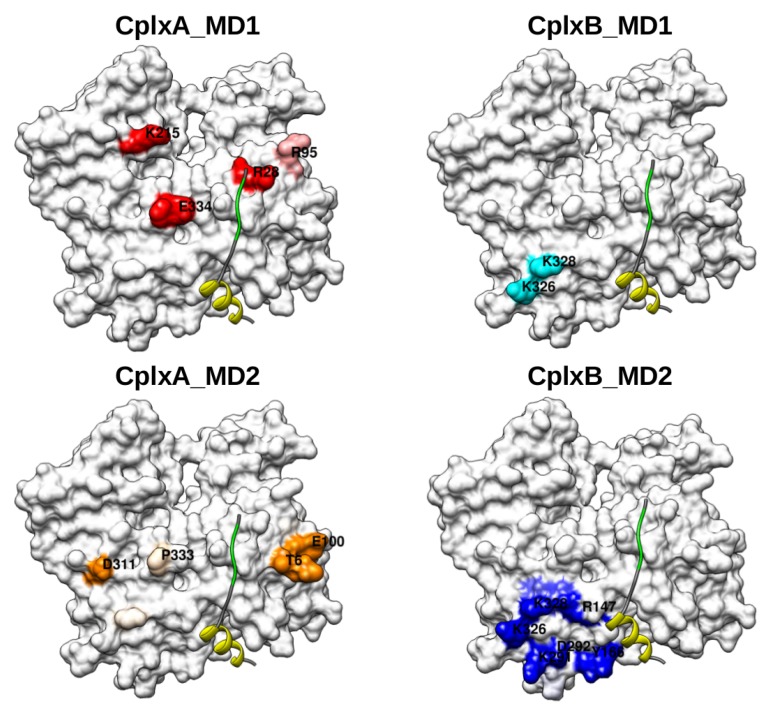
Actin residues distant by less than 4 Å from N-WASP segments ${}^{28}$NSRPVS${}^{33}$ or ${}^{56}$GQESTP${}^{61}$ in CplxA_MD1 (red), CplxA_MD2 (orange), CplxB_MD1 (cyan), and CplxB_MD2 (blue). As a reference, yellow and green ribbons represent the helical region and the conserved sequence LKSV of the second WH2 motif observed in structure 2VCP [27].

The actin residues that have high probabilities to be contacted by regions ${}^{28}$NSRPVS${}^{33}$ and ${}^{56}$GQESTP${}^{61}$ are displayed in Figure 8. In both simulations of CplxB, segment ${}^{56}$GQESTP${}^{61}$ was mostly found in contact with residues of the actin subdomain 3. In CplxB_MD1, this might be the reason the conserved segment ${}^{50}$LKSV${}^{53}$ cannot reach its cognate binding site on actin. In CplxB_MD2, this is probably because the helix 37–46 slid toward the bottom of actin and that segment ${}^{50}$LKSV${}^{53}$ is improperly located between actin subdomains 1 and 3 (Figure 6). Strikingly, in simulations of CplxA in which the helical segment 9–18 and conserved sequence ${}^{22}$LKKV${}^{25}$ are both satisfactorily positioned on actin’s surface, the region ${}^{28}$NSRPVS${}^{33}$ is observed to contact several separated patches on actin’s surface, mainly located on subdomains 2 and 4. This might explain why these disordered regions cannot crystallize in one homogeneous conformation and, therefore, are not visible in most crystallographic actin–WH2 complexes.

### 2.3. Dimeric Actin–Domain V Encounter Complexes Generated by Docking Calculations

As reported in the literature, a tandem of WH2 motifs, such as N-WASP domain V, can form a ternary complex with two actin molecules [26,32]. Rebowski et al. notably reported a 2:1 actin–domain V complex, in which two actins are assembled into a longitudinal filament-like dimer (PDB structure 3M3N) [26]. In this section, we investigate the early steps of formation of these ternary encounter complexes. As for actin monomer, we blindly docked the 527 most populated clusters of the MD-derived N-WASP domain V conformational ensemble [29], but here, onto the longitudinal actin dimer structure extracted from the PDB file 3M3N [26]. It should be noted that each chain of the 3M3N dimer is structurally very similar to actin in 2VCP (RMSD over Cα atoms being equal to 0.99 and 0.66 Å for chain A and B, respectively). Moreover, unlike in 2VCP structure, both chains of actin dimer 3M3N lack the coordinates of their last residue F375. A total number of 754,118 complex structures were generated. The likeliness of these complexes was evaluated with the scoring function 2/3B${}^{best}$ InterEvScore [30]. We delineated the 1% complexes (that is 7540 conformers) having the highest 2/3B${}^{best}$ score as the most probable actin dimer-domain V structures. As for actin monomer, when compared to the 527 cluster representative structures, the domain V conformations that are retrieved in the most probable complexes with actin dimer are in average more compact as indicated by the radius of gyration distributions (Figure S3). The dimeric state of actin did not favor the binding of extended conformations of domain V.

**Figure 9:**
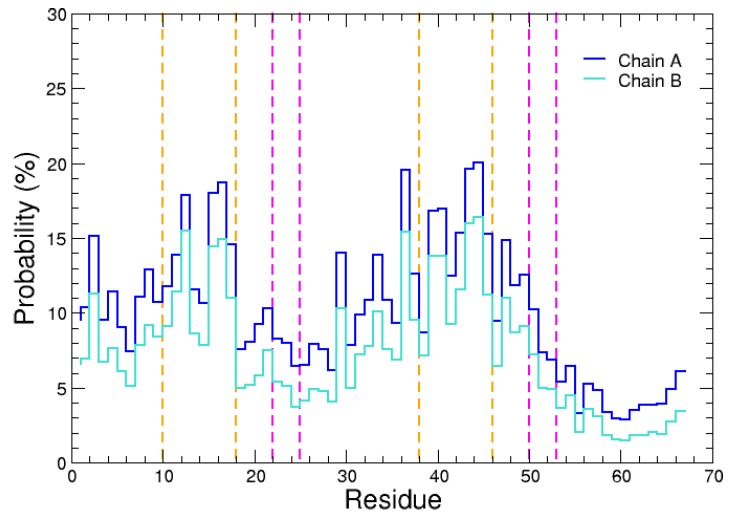
Probability of the N-WASP domain V residues to be distant by less than 4 Å from actin dimer. Orange and magenta dashed lines indicate the N-WASP regions in α-helix (as revealed by the X-ray structure 2VCP [27]) and the consensus sequences “LKKV” [14,31], respectively.

We then analyzed the probability of domain V residues to be in contact with each chain of actin dimer. We observed again that actin preferentially recognizes the domain V regions ${}^{9}$KAALLDQIRE${}^{18}$ and ${}^{37}$RDALLDQIRQ${}^{46}$, with a similar pattern as for actin monomer (compare Figure 9 with Figure 1), indicating that the N-WASP recognized regions are rather in (partial) α-helical structures. It is also confirmed that the conserved sequences ${}^{22}$LKKV${}^{25}$ and ${}^{50}$LKSV${}^{53}$ have low probability to be contacted by actin dimer in the encounter complexes, suggesting again that they should move and anchor to the actin’s surface after the recognition of the previously mentioned regions 9–18 and 37–46.

**Figure 10:**
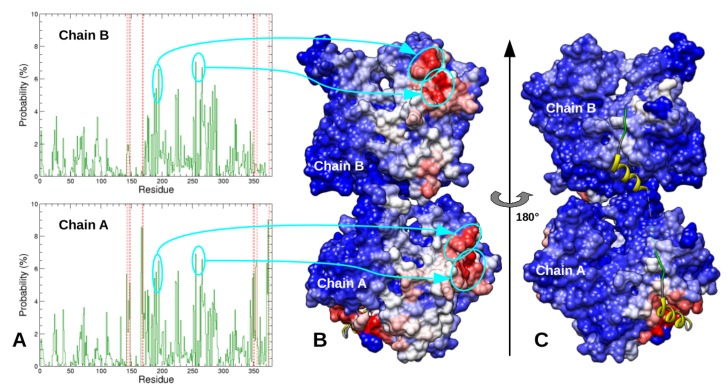
(**A**) Residue-specific probability of actin dimer chain A (bottom) and chain B (top) to be distant by less than 4 Å from N-WASP domain V regions 9–18 or 37–46 in the ensemble of 7540 ternary complexes generated by docking. Red dashed lines indicate the actin residues in contact with the N-WASP helical segment in the X-ray structure 2VCP [27]. (**B**,**C**) Back and front views of the actin dimer surface colored proportionally to the previous probabilities. Blue, white, and red colors indicate actin residues with low, intermediate, and high probabilities to be contacted by N-WASP domain V regions 9–18 or 37–46, respectively. As a reference, yellow and green ribbons represent helical regions and consensus sequences “LKKV” of the two WH2 motifs observed in the X-ray structure 3M3N [26].

Finally, we determined the preferential location of the domain V regions 9–18 and 37–46 on actin dimer surface by computing over the 7540 most probable complexes the probability that actin residues are contacted by one of these segments (Figure 10). The N-WASP regions 9–18 and 37–46 can be retrieved in the cognate binding site of actin chain A but not of chain B. The presence of chain A at the bottom of chain B probably hinders the approach and accommodation of domain V in the binding site of chain B. As for actin monomer, we also observed that N-WASP segments 9–18 and 37–46 can bind to other patches of the actin’s surface with high probability, notably at residues K191, E195, R256 and F266 which are located at the top of the back of actin dimer (Figure 10). It is not clear for us if these non-productive associations are artifacts or not. Nevertheless, since the consensus sequences “LKKV” have low probabilities to contact actin, large conformational changes of domain V are likely to occur after the formation of the encounter complexes. Only a productive encounter complex in which the cognate binding site of actin accommodates N-WASP segment 9–18 or 37–46 will be able to form the correct quaternary structure.

**Figure 11:**
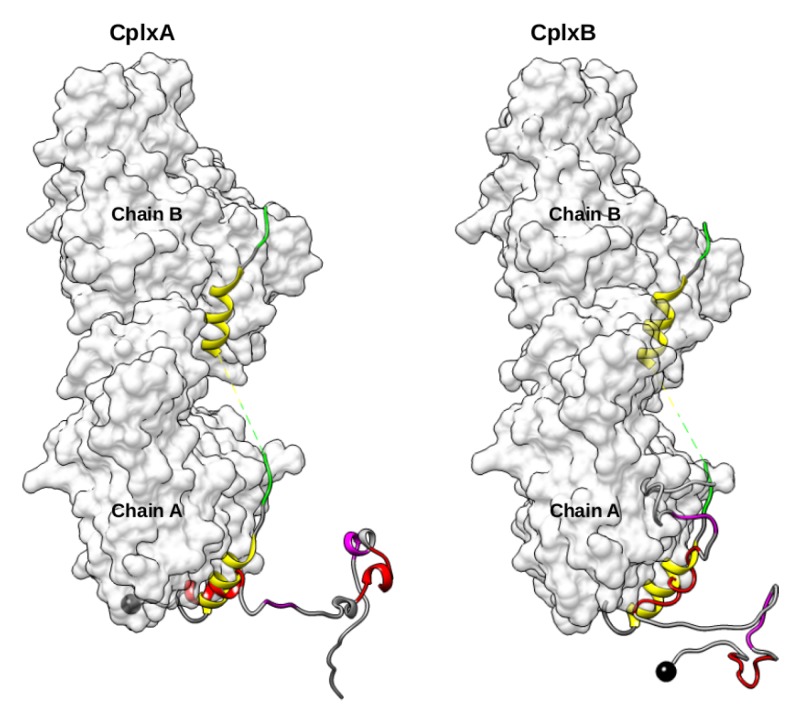
Side view of the two best 2:1 actin–domain V encounter complexes with N-WASP segment 9–18 (**left**) or 37–46 (**right**) located and oriented as in structure 3M3N. Black balls are N-terminal Cα-atoms of domain V. Red and magenta ribbons represent its regions 9–18 or 37–46 and consensus sequences “LKKV”, respectively. As a reference, yellow and green ribbons indicate the helical and LKKV regions of domain V in 3M3N.

These productive actin–domain V encounter complexes were identified among the 7540 most probable complexes as those with segment 9–18 or 37–46 making contacts to at least 6 over the 8 hot-spot residues of 3M3N actin chain A (Y143, G146, T148, G168, Y169, L349, T351, and M355), and correctly oriented so that the conserved sequence ${}^{22}$LKKV${}^{25}$ or ${}^{50}$LKSV${}^{53}$ can reach their cognate binding site. We found 10 and 13 productive encounter complexes in which N-WASP segments 9–18 and 37–46 are bound to actin chain A, respectively (Tables S4 and S5). The two complexes for which the regions 9–18 or 37–46 have the lowest RMSD relative to structure 3M3N are displayed in Figure 11. In all found productive encounter complexes, regions ${}^{22}$LKKV${}^{25}$ or ${}^{50}$LKSV${}^{53}$ are detached from actin, and actin chain B is not contacted by other parts of N-WASP domain V. The presence of chain B in the actin dimer does not seem to influence the recognition of N-WASP segments 9–18 or 37–46 by actin chain A. Besides, several representative structures of domain V conformational ensemble (clusters 105, 145, 230, 333, 407, and 411) were retrieved in the most probable encounter complexes on both the monomeric (2VCP) and dimeric (3M3N) states of actin. Nevertheless, as previously seen, the subsequent binding of residues ${}^{22}$LKKV${}^{25}$ or ${}^{50}$LKSV${}^{53}$ to actin was not persistent in our MD simulation of complexes with actin monomer, but this association might be stabilized by the presence of a second chain in complexes with actin dimer. This hypothesis can be assessed using extensive MD simulations. Unfortunately, our limited computational resources for this project did not allow us to perform these calculations.

## 3. Discussion

The characterization of the early events of protein–protein recognitions involving intrinsically disordered proteins is important for better understanding the molecular bases of regulation and signaling processes occurring in cells. This task is very challenging using current experimental techniques and can be fruitfully complemented by molecular modeling. However, MD simulations of encounter complexes starting from separated proteins are computationally very demanding and require extremely long trajectories in cases of IDPs. In this study, we propose a less expensive approach consisting, first, in discretizing the IDP large conformational ensemble into representative structures of the most populated clusters; secondly, in generating the protein–protein encounter complexes by rigid coarse-grained protein–protein docking; and, finally, in performing MD calculations of few selected productive complex conformations.

This approach was used to study the recognition by actin of the two WH2 motifs of N-WASP domain V, which is largely disordered in free state. Several crystallographic structures of actin–WH2 motif complexes show that the WH2 motif N-terminal part is folded into an amphiphilic α-helix located in a cleft at the bottom of actin, and that its consensus sequence “LKKV” has a rather extended conformation lying on the actin front surface (Figure 2 and Figure 6). The pathway leading to these bound states remains largely unknown, especially in the case of tandems of WH2 motifs which bind two actins.

Previously, we identified several structures with transient α-helices at regions 9–18 and 37–46 in the unbound domain V conformational ensemble [29]. Our present docking calculations showed that these two regions are effectively preferential binding sites for actin (Figure 1). Our results also suggest that conformations with regions 9–18 or 37–46 completely structured in α-helix are not preferably recognized, but less folded conformations can be equally accommodated in the cognate binding site on actin (Tables S2–S5). Knowing the binding location on actin’s surface of the conserved segments ${}^{22}$LKKV${}^{25}$ or ${}^{50}$LKSV${}^{53}$, it is apparent that non-specific association and orientation of regions 9–18 and 37–46 on actin’s surface cannot produce the observed quaternary structure of actin–WH2 motif complexes. Our MD simulations of a productive encounter complex even showed that, when the recognized helical region 37–46 of N-WASP is initially correctly located and oriented in the actin cognate binding site, a slight displacement of this region toward the bottom of actin prevents the segment ${}^{50}$LKSV${}^{53}$ to reach and bind its specific site on actin (simulation CplxB_MD2).

In our modeling procedure, it could be noted that only the 7030 encounter complexes with the highest 2/3B${}^{best}$ score among the 702,920 generated by docking were deemed as probable and subsequently analyzed. Although this limited number could lead to possible missed relevant structures, it is much larger than the number of docking solutions that are usually analyzed to find near-native protein–protein interfaces (up to 1000) [30]. This provides reasonable confidence that our modeling generated relevant quaternary structures. Besides, the 7030 analyzed structures can be considered as representative of both the productive and non-productive encounter complexes (Figure 2), as they probably appear in vitro or in vivo. Strikingly, in all productive encounter complexes, the consensus sequence “LKKV” of WH2 motifs is found distant from actin’s surface (Figure 3). This indicates that large amplitude motions of these segments are likely to occur in a second step to enable the formation of the final quaternary structure, as illustrated in our MD simulations of CplxA (Figure 5 and Figure 7). Thus, we think that our modeling study has allowed going beyond the prediction of the actin–N-WASP complex quaternary structure and has also gained insight into its mechanism of formation. To sum up, our study of actin monomer recognition by N-WASP domain V indicates that actin first binds domain V regions 9–18 or 37–46 which are partially folded into amphiphilic helical structures, mainly through hydrophobic interactions. Then, the charged segments ${}^{22}$LKKV${}^{25}$ or ${}^{50}$LKSV${}^{53}$, driven by electrostatic forces, move and attach to their cognate site on actin’s surface.

**Figure 12:**
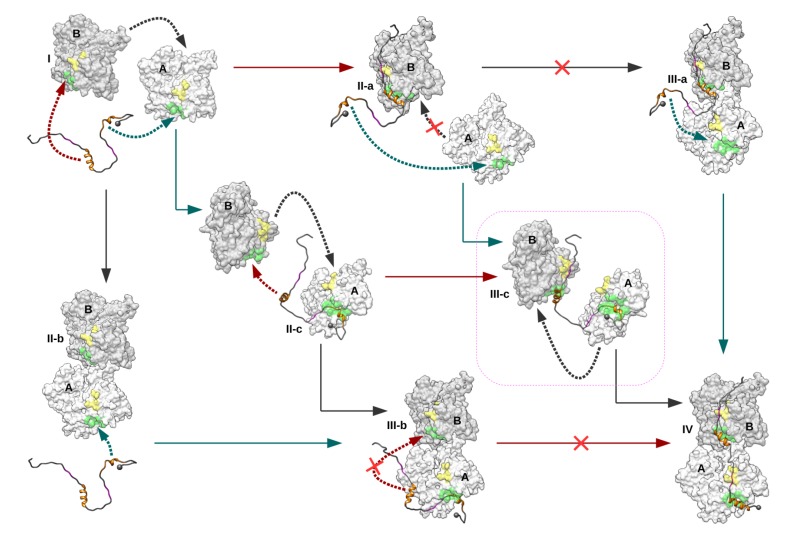
Possible pathways toward the formation of a 2:1 actin–domain V complex with a longitudinal actin dimer as observed in 3M3N. Starting from two actin chains and one N-WASP domain V (State I), three possible binary encounter complexes can be formed (States II-a, II-b, and II-c), leading to three possible intermediate ternary complexes (States III-a, III-b, and III-c) just before the final structure (State IV). Cyan and red arrows indicate the binding of the N-WASP first and second WH2 motif to actin chain A and B, respectively. Dark grey arrows represent the binding of two actins into a longitudinal dimer.

When the binding of domain V to a longitudinal actin dimer was considered, our docking calculations showed that N-WASP helical regions 9–18 and 37–46 can bind their cognate binding sites, but preferentially on actin chain A, the access of the specific binding site on chain B being more restricted (Figure 10). Nevertheless, this result might depend on the quaternary structure of the actin dimer, particularly on the actin–actin interface, which can significantly vary, as observed in various crystallographic structures of actin oligomers (3M3N [26], 4JHD [32], and 6FHL [33]). All together, our results allow us to propose the following model for the early events of association of N-WASP domain V to two actins and the formation of a ternary complex with a longitudinal filament-like actin dimer, as observed in structure 3M3N (Figure 12): From isolated actin chains and N-WASP domain V, three possible binary complexes can be formed (States II-a, II-b, and II-c). In State II-a, the second WH2 motif attached to actin chain B prevents the approach and binding of chain A [11,15,34] and thus disfavors the formation of intermediate State III-a. When the actin dimer is already formed, our docking calculations indicate that the binding of N-WASP second WH2 motif to actin chain B is not favorable. Thus, the direct formation of the ternary State III-a from a preformed actin dimer or the evolution of intermediate State III-b toward the final complex are very unlikely. These considerations imply that the final state is likely formed through an intermediate ternary complex in which the two WH2 motifs are bound to two loosely interacting actin chains (State III-c). Then, this highly flexible assembly evolves toward the final state through the association of the two actin chains into a longitudinal dimer. This model suggests that the binding of N-WASP domain V to an actin dimer would not be a cooperative process, in line with fluorescence titration experiments reported by Gaucher et al. [27].

During this process, it is not clear whether the binding of the conserved sequences ${}^{22}$LKKV${}^{25}$ and ${}^{50}$LKSV${}^{53}$ to their cognate sites occurs before the formation of the longitudinal dimer. In crystallographic structure 2VCP, the four residues ${}^{50}$LKSV${}^{53}$ are found attached to the actin’s surface, but our MD simulations in explicit water indicate that this binding is rather transient in 1:1 actin–domain V complexes. We speculate that the interactions between the consensus sequences and actin might guide the dynamics of dimerization into longitudinal assemblies. All together, our model for the early events of domain V association to two actins might explain how the two WH2 motifs of N-WASP favor the formation of longitudinal filament-like conformation of actin dimer and why they induce more rapid actin polymerization than proteins of the WASP family with only one WH2 motif [28].

## 4. Methods

### 4.1. Conformational Clustering

The conformational ensemble of the studied construct encompassing N-WASP domain V and previously generated by MD simulations with the Amber-03ws force field [29] was clustered with the GROMACS tool *gmx cluster* using the *gromos* method [35] and a RMSD cutoff of 0.5 nm (computed over the mainchain atoms). We obtained 2467 clusters and decided, for subsequent protein–protein docking calculations, to keep only the 527 most populated ones, which represent 50% of the 40,000 conformations sampled by MD simulations. To verify that the 527 clusters are representative of the overall conformational ensemble, we compared the residue probabilities to be in α-helix and the distributions of gyration averaged over the 40,000 conformations or the 527 cluster structures. As shown in Figure S2, the probabilities to form α-helices of the 527 clusters and of the whole conformational ensemble are almost identical, and the protein radius of gyration has similar distributions when computed over the sub-ensemble of representative structures or over the 40,000 conformations. This indicates that the selected 527 conformers are locally and globally representative of the whole conformational ensemble of N-WASP domain V.

### 4.2. Protein–Protein Docking

The 527 representative conformations of N-WASP domain V were docked into two crystallographic structures of actin (PDB ID: 2VCP [27] and 3M3N [26]), using the molecular modeling library PTools [36]. This toolbox performs rigid-body docking of coarse-grained proteins by multiple energy minimizations, starting from regularly distributed initial positions and orientations of the ligand around the receptor surface. It should be emphasized that no conformational change was allowed during these docking calculations for both protein partners, notably the intrinsically disordered domain V. The energy function minimized here is the physics-based pairwise protein–protein interaction energy SCORPION [37,38]. Then, to better discriminate the near-native interface between actin and domain V, the complexes previously generated with PTools were rescored using a knowledge-based scoring function which additionally takes into account three-body interactions. We used in this study the 2/3B${}^{best}$ InterEvScore, without any evolutionary information from the actin or N-WASP domain V sequences [30].

The performance of 2/3B${}^{best}$ InterEvScore was positively evaluated on an ensemble of 131 protein–protein complexes which, as far as we know, did not include IDP case [30]. Thus, to assess the validity of our approach to study the actin–domain V recognition, we performed the redocking of the folded segment 433–451 of N-WASP domain V into actin structure 2VCP [27] and checked if the X-ray structure of the complex can be retrieved. The results of this test are reported in Figure S4, which displays the actin–ligand interaction 2/3B${}^{best}$ score as a function of the RMSD relative to the peptide conformation in the crystallographic structure. It can be seen that the coarse-grained protein–protein redocking is able to retrieve the experimental structure with a RMSD calculated over the Cα atoms of only 0.5 Å. In this particular case, the modeled complex structure, which is the closest to the experimental one is ranked first (the higher is the score, the more native-like is the interface). This benchmark led us to adopt this two-step approach consisting in generating complex structures with PTools and rescoring them with InterEvScore.

### 4.3. MD Simulations

From the docking results, several probable structures of the actin–domain V complex were selected and submitted to extensive MD simulations performed with the GROMACS software (versions 5.0.2 and 2016.1) [39]. Each selected complex initial conformation was put and solvated in a dodecahedral rhombic box of 14.0 nm edge, then neutralized by adding 175 sodium and 176 chloride ions to reach the salt concentration of 150 mM. The non-bonded interactions were treated using the smooth PME method [40] for the electrostatic terms and a cutoff distance of 1.2 nm for the van der Waals potentials. The solute and water covalent bond lengths were kept constant using the LINCS [41] and SETTLE [42] algorithms, respectively, allowing to integrate the equations of motion with a 2 fs time step. All simulations were run in the NPT ensemble, at T = 310 K and P = 1 bar, using the Nose–Hoover and Parrinello–Rahman algorithms [43,44,45] with the time coupling constants $\tau_{T}=0.5$ ps and $\tau_{P}=2.5$ ps.

In our previous study of the free state N-WASP domain V, short preliminary MD simulations indicated that the force field AMBER-03w [46] combined with the modified water model TIP4P/2005s [47] (a combination referred to as A03ws) allowed correctly exploring the protein conformational space. For consistency, we kept this force field for the study of its complex with actin. Each selected complex was submitted to about 350 ns MD simulations within the general conditions previously described. Data collected every 20 ps were kept for subsequent analyses. The latter were made using mostly the GROMACS tools, such as *gmx mindist* or *gmx cluster* for computing specific distances or structural clusters, respectively. The program STRIDE [48] was used to assign secondary structures to the protein residues.

## Supplementary Materials

The following are available online at https://www.mdpi.com/1422-0067/20/18/4493/s1. Table S1: List of proteins with WH2 motifs which were co-crystallized with actin; Figure S1: Alignment of the studied construct sequence with those of N-WASP in 2VCP and 3M3N structures; Figure S2: N-WASP domain V residue probabilities to be in α-helix and distributions of gyration of the 7030 conformations in the most probable 1:1 actin–domain V complexes; Table S2: Most probable 1:1 actin–domain V encounter complexes in which domain V segment 9–18 is in contact with at least six over nine actin hot-spot residues; Table S3: Most probable 1:1 actin–domain V encounter complexes in which domain V segment 37–46 is in contact with at least six over nine actin hot-spot residues; Figure S3: N-WASP domain V residue probabilities to be in α-helix and distributions of gyration of the 7540 conformations in the most probable 2:1 actin–domain V complexes; Table S4: Most probable 2:1 actin–domain V encounter complexes in which segment 9–18 is in contact with at least six over eight actin hot-spot residues; Table S5: Most probable 2:1 actin–domain V encounter complexes in which segment 37–46 is in contact with at least six over eight actin hot-spot residues; Figure S4: 2/3B${}^{best}$ score of N-WASP segment 433–451 redocked into actin as a function of the ligand RMSD relative to the conformation found in structure 2VCP.

## Abbreviations

The following abbreviations are used in this manuscript:

IDP	Intrinsically Disordered Protein
IDR	Intrinsically Disordered Region
PDB	Protein Data Bank
N-WASP	Neural Wiskott–Aldrich Syndrome Protein
MoRF	Molecular Recognition Feature
NMR	Nuclear Magnetic Resonance
SAXS	Small-Angle X-ray Scattering
MD	Molecular Dynamics
RMSD	Root Mean Square Deviation

## References

[1] Wright P.E., Dyson H.J. (1999). Intrinsically unstructured proteins: Re-assessing the protein structure-function paradigm. J. Mol. Biol..

[2] Dunker A.K., Lawson J.D., Brown C.J., Williams R.M., Romero P., Oh J.S., Oldfield C.J., Campen A.M., Ratliff C.M., Hipps K.W. (2001). Intrinsically disordered protein. J. Mol. Graph. Model..

[3] Dyson H.J., Wright P.E. (2005). Intrinsically unstructured proteins and their functions. Nat. Rev. Mol. Cell Biol..

[4] Dunker A.K., Silman I., Uversky V.N., Sussman J.L. (2008). Function and structure of inherently disordered proteins. Curr. Opin. Struct. Biol..

[5] Zea D.J., Monzon A.M., Gonzalez C., Fornasari M.S., Tosatto S.C.E., Parisi G. (2016). Disorder transitions and conformational diversity cooperatively modulate biological function in proteins. Protein Sci..

[6] Oldfield C.J., Cheng Y., Cortese M.S., Romero P., Uversky V.N., Dunker A.K. (2005). Coupled Folding and Binding with *α*-Helix-Forming Molecular Recognition Elements. Biochemistry.

[7] Mohan A., Oldfield C.J., Radivojac P., Vacic V., Cortese M.S., Dunker A.K., Uversky V.N. (2006). Analysis of Molecular Recognition Features (MoRFs). J. Mol. Biol..

[8] Vacic V., Oldfield C.J., Mohan A., Radivojac P., Cortese M.S., Uversky V.N., Dunker A.K. (2007). Characterization of Molecular Recognition Features, MoRFs, and Their Binding Partners. J. Proteome Res..

[9] Cheng Y., Oldfield C.J., Meng J., Romero P., Uversky V.N., Dunker A.K. (2007). Mining *α*-helix-forming molecular recognition features with cross species sequence alignments. Biochemistry.

[10] Lee C., Kalmar L., Xue B., Tompa P., Daughdrill G.W., Uversky V.N., Han K.H. (2014). Contribution of proline to the pre-structuring tendency of transient helical secondary structure elements in intrinsically disordered proteins. Biochim. Biophys. Acta Gen. Subj..

[11] Carlier M.F., Husson C., Renault L., Didry D., Jeon K.W. (2011). Chapter Two–Control of Actin Assembly by the WH2 Domains and Their Multifunctional Tandem Repeats in Spire and Cordon-Bleu. International Review of Cell and Molecular Biology.

[12] Derry J.M.J., Ochs H.D., Francke U. (1994). Isolation of a novel gene mutated in Wiskott-Aldrich syndrome. Cell.

[13] Palma A., Ortega C., Romero P., Garcia-V A., Roman C., Molina I., Santamaria M. (2004). Wiskott-Aldrich syndrome protein (WASp) and relatives: A many-sided family. Immunologia.

[14] Chereau D., Kerff F., Graceffa P., Grabarek Z., Langsetmo K., Dominguez R. (2005). Actin-bound structures of Wiskott-Aldrich syndrome protein (WASP)-homology domain 2 and the implications for filament assembly. Proc. Natl. Acad. Sci. USA.

[15] Renault L., Deville C., van Heijenoort C. (2013). Structural features and interfacial properties of WH2, *β*-thymosin domains and other intrinsically disordered domains in the regulation of actin cytoskeleton dynamics. Cytoskeleton.

[16] Kiefhaber T., Bachmann A., Jensen K.S. (2012). Dynamics and mechanisms of coupled protein folding and binding reactions. Curr. Opin. Struct. Biol..

[17] Liu X., Chen J., Chen J. (2019). Residual Structure Accelerates Binding of Intrinsically Disordered ACTR by Promoting Efficient Folding upon Encounter. J. Mol. Biol..

[18] Kozakov D., Li K., Hall D.R., Beglov D., Zheng J., Vakili P., Schueler-Furman O., Paschalidis I.C., Clore G.M., Vajda S. (2014). Encounter complexes and dimensionality reduction in protein–protein association. eLife.

[19] Pollard T.D., Borisy G.G. (2003). Cellular Motility Driven by Assembly and Disassembly of Actin Filaments. Cell.

[20] Takenawa T., Suetsugu S. (2007). The WASP–WAVE protein network: connecting the membrane to the cytoskeleton. Nat. Rev. Mol. Cell Biol..

[21] Miki H., Miura K., Takenawa T. (1996). N-WASP, a novel actin-depolymerizing protein, regulates the cortical cytoskeletal rearrangement in a PIP2-dependent manner downstream of tyrosine kinases. Embo J..

[22] Prehoda K.E., Scott J.A., Mullins R.D., Lim W.A. (2000). Integration of Multiple Signals Through Cooperative Regulation of the N-WASP-Arp2/3 Complex. Science.

[23] Fawcett J., Pawson T. (2000). N-WASP Regulation—The Sting in the Tail. Science.

[24] Luan Q., Zelter A., MacCoss M.J., Davis T.N., Nolen B.J. (2018). Identification of Wiskott-Aldrich syndrome protein (WASP) binding sites on the branched actin filament nucleator Arp2/3 complex. Proc. Natl. Acad. Sci. USA.

[25] Dominguez R. (2009). Actin filament nucleation and elongation factors— Structure–function relationships. Crit. Rev. Biochem. Mol. Biol..

[26] Rebowski G., Namgoong S., Boczkowska M., Leavis P.C., Navaza J., Dominguez R. (2010). Structure of a Longitudinal Actin Dimer Assembled by Tandem W Domains: Implications for Actin Filament Nucleation. J. Mol. Biol..

[27] Gaucher J.F., Maugé C., Didry D., Guichard B., Renault L., Carlier M.F. (2012). Interactions of Isolated C-terminal Fragments of Neural Wiskott-Aldrich Syndrome Protein (N-WASP) with Actin and Arp2/3 Complex. J. Biol. Chem..

[28] Yamaguchi H., Miki H., Suetsugu S., Ma L., Kirschner M.W., Takenawa T. (2000). Two tandem verprolin homology domains are necessary for a strong activation of Arp2/3 complex-induced actin polymerization and induction of microspike formation by N-WASP. Proc. Natl. Acad. Sci. USA.

[29] Chan-Yao-Chong M., Deville C., Pinet L., van Heijenoort C., Durand D., Ha-Duong T. (2019). Structural Characterization of N-WASP Domain V Using MD Simulations with NMR and SAXS Data. Biophys. J..

[30] Andreani J., Faure G., Guerois R. (2013). InterEvScore: a novel coarse-grained interface scoring function using a multi-body statistical potential coupled to evolution. Bioinformatics.

[31] Kollmar M., Lbik D., Enge S. (2012). Evolution of the eukaryotic ARP2/3 activators of the WASP family: WASP, WAVE, WASH, and WHAMM, and the proposed new family members WAWH and WAML. BMC Res. Notes.

[32] Chen X., Ni F., Tian X., Kondrashkina E., Wang Q., Ma J. (2013). Structural Basis of Actin Filament Nucleation by Tandem W Domains. Cell Rep..

[33] Merino F., Pospich S., Funk J., Wagner T., Küllmer F., Arndt H.D., Bieling P., Raunser S. (2018). Structural transitions of F-actin upon ATP hydrolysis at near-atomic resolution revealed by cryo-EM. Nat. Struct. Mol. Biol..

[34] Hertzog M., van Heijenoort C., Didry D., Gaudier M., Coutant J., Gigant B., Didelot G., Préat T., Knossow M., Guittet E. (2004). The *β*-Thymosin/WH2 Domain: Structural Basis for the Switch from Inhibition to Promotion of Actin Assembly. Cell.

[35] Daura X., Gademann K., Jaun B., Seebach D., van Gunsteren W.F., Mark A.E. (1999). Peptide Folding: When Simulation Meets Experiment. Angew. Chem. Int. Ed..

[36] Saladin A., Fiorucci S., Poulain P., Prévost C., Zacharias M. (2009). PTools: An opensource molecular docking library. BMC Struct. Biol..

[37] Basdevant N., Borgis D., Ha-Duong T. (2007). A Coarse-Grained Protein–Protein Potential Derived from an All-Atom Force Field. J. Phys. Chem. B.

[38] Basdevant N., Borgis D., Ha-Duong T. (2013). Modeling Protein–Protein Recognition in Solution Using the Coarse-Grained Force Field SCORPION. J. Chem. Theory Comput..

[39] Abraham M.J., Murtola T., Schulz R., Pall S., Smith J.C., Hess B., Lindahl E. (2015). GROMACS: High performance molecular simulations through multi-level parallelism from laptops to supercomputers. SoftwareX.

[40] Essmann U., Perera L., Berkowitz M.L., Darden T., Lee H., Pedersen L.G. (1995). A smooth particle mesh Ewald method. J. Chem. Phys..

[41] Hess B. (2008). P-LINCS: A Parallel Linear Constraint Solver for Molecular Simulation. J. Chem. Theory Comput..

[42] Miyamoto S., Kollman P.A. (1992). SETTLE: An analytical version of the SHAKE and RATTLE algorithm for rigid water models. J. Comput. Chem..

[43] Nosé S. (1984). A unified formulation of the constant temperature molecular dynamics methods. J. Chem. Phys..

[44] Hoover W.G. (1985). Canonical dynamics: Equilibrium phase-space distributions. Phys. Rev. A.

[45] Parrinello M., Rahman A. (1981). Polymorphic transitions in single crystals: A new molecular dynamics method. J. Appl. Phys..

[46] Best R.B., Mittal J. (2010). Protein Simulations with an Optimized Water Model: Cooperative Helix Formation and Temperature-Induced Unfolded State Collapse. J. Phys. Chem. B.

[47] Best R.B., Zheng W., Mittal J. (2014). Balanced Protein–Water Interactions Improve Properties of Disordered Proteins and Non-Specific Protein Association. J. Chem. Theory Comput..

[48] Heinig M., Frishman D. (2004). STRIDE: A web server for secondary structure assignment from known atomic coordinates of proteins. Nucleic Acids Res..

