# Supplementary material for: Investigation into Early Steps of Actin Recognition by the Intrinsically Disordered N-WASP Domain V
Source: Int J Mol Sci. 2019 Sep 11;20(18):4493. doi: 10.3390/ijms20184493 (PMC6770570; doi:10.3390/ijms20184493)
Supplement: Supplementary file 1 [file ijms-20-04493-s001.pdf]

Supplementary Materials:

Investigation into early steps of actin recognition by the intrinsically disordered N-WASP domain V

Maud Chan-Yao-Chong [1,2], Dominique Durand [2], and Tâp Ha-Duong [1,*]

Protein Name	Uniprot ID	PDB ID	Actin-protein stoichiometry	Co-crystallized protein residues	Visible residues	Ref.
**WASP**	P42768	2A3Z	1:1	430-458	431-447	[1]
**WAVE2**	Q9Y6W5	2A40	1:1	432-463	433-454	[1]
**WIP**	O43516	2A41	1:1	29-60	29-60	[1]
**MIM**	O43312	2D1K	1:1	724-755	725-753	[2]
**N-WASP**	O00401	2VCP	1:1	392-484	433-452	[3]
	Q91YD9	3M3N	2:1	397-497	397-418; 429-446	[4]
**Cordon-Bleu**	Q5NBX1	4JHD	2:1	1176-1337	1184-1264	[5]
		5YPU	1:1	1184-1205	1184-1205	[6]
**VopL**	Q87GE5	3M1F	1:1	130-160	130-151	[4]
**Spire**	Q9U1K1	3MMV	1:1	Undetermined	Undetermined	[7]
		3MN5	1:1	448-485	461-480	[7]
		3MN6	1:1	Undetermined	Undetermined	[7]
		3MN7	1:1	382-480	461-480	[7]
		3MN9	1:1	Undetermined	Undetermined	[7]
		3UE5	1:1	423-488	457-481	[8]
		4EFH	1:1	423-488	461-480	[8]

Table S1. List of proteins with WH2 motifs which were co-crystallized with actin. Four proteins of the WH2 family have one WH2 motif (residues indicated in brackets): WASP (Wiskott-Aldrich Syndrome Protein; 430-461), WAVE2 (Wiskott-Aldrich syndrome protein family member 2; 436-470), WIP (WASP-Interacting Protein; 32-64), and MIM (Missing In Metastasis; 727-755). N-WASP (Neural Wiskott-Aldrich Syndrome Protein) has a tandem of two WH2 motifs (405-433; 434-464) and was co-crystallized into a 1:1 (2VCP) and a 2:1 (3M3N) actin-protein complexes. Cordon-bleu and VopL (Vibrio parahaemolyticus) are two proteins with three WH2 motifs (segments 1185-1224; 1225-1265; 1313-1337 for Cordon-Bleu and 134-160; 164-187; 204-223 for VopL). Finally, the protein Spire contains the largest number of WH2 modules with four WH2 motifs (371-398; 399-431; 432-462; 463-485). It could be noted that a 19-residues segment of Spire is visible in 3MMV, 3MN6, and 3MN9 co-crystals, but its sequence was not determined.

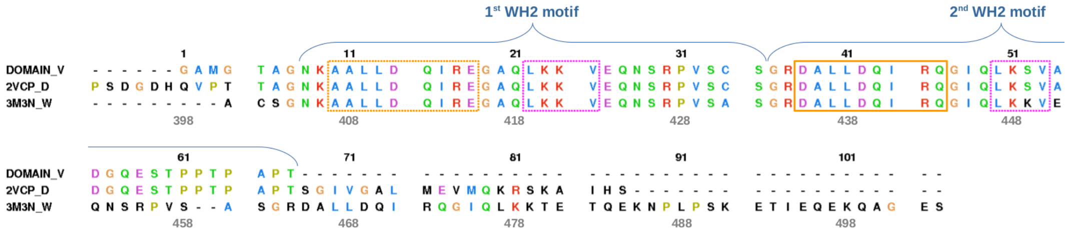

Figure S1. Alignment of the construct sequence used in the present study (DOMAIN_V) with those of N-WASP domain VC co-crystallized with one actin (2VCP_D) by Gaucher *et al.* [3] and of a WH2 tandem co-crystallized with a longitudinal actin dimer (3M3N_W) by Rebowski *et al.* [4]. Orange and magenta solid box indicate the helical region and the consensus sequence "LKKV" of the second WH2 motif present in the X-ray structure 2VCP. Orange and magenta dashed box indicate the homologous regions in the first WH2 motif.

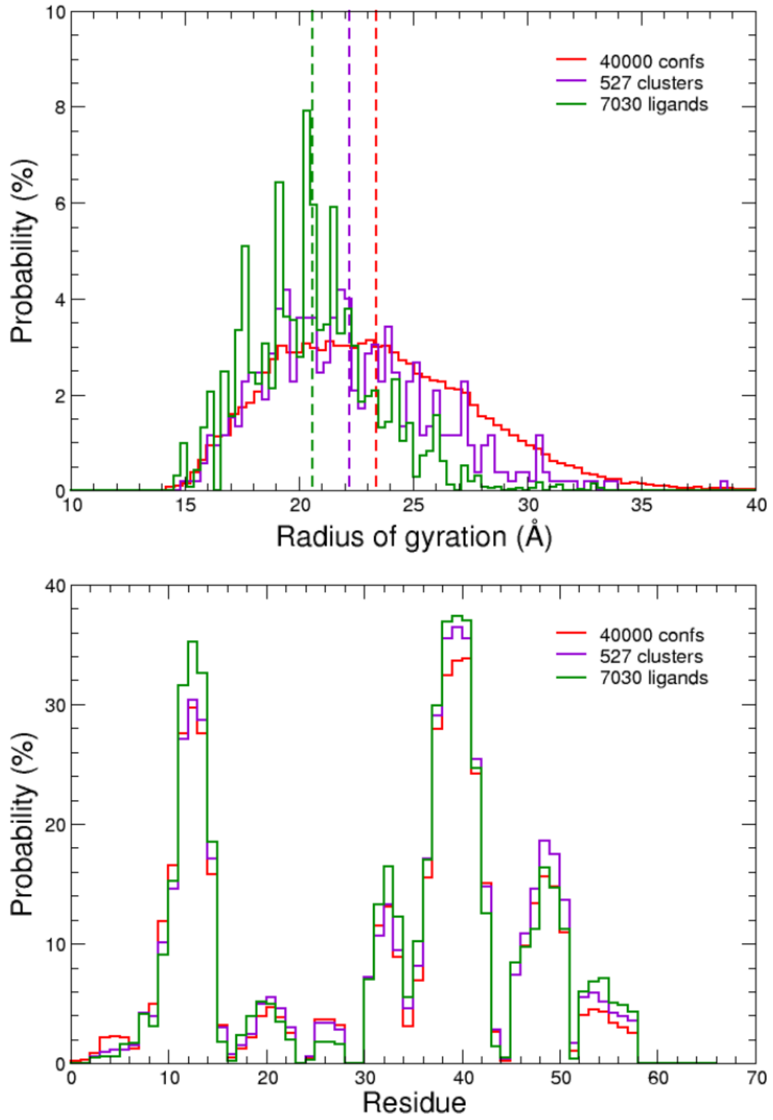

Figure S2. Comparison of N-WASP domain V residue probabilities to be in α-helix (bottom) and distributions of gyration (top) between the ensemble of initial 40 000 conformations (red), the 527 cluster representative structures (violet), and the 7 030 conformers in the most probable 1:1 actin-domain V complexes (green). Vertical dashed lines indicate average values of radius of gyration for the three conformational ensembles.

Cluster ID	Pose ID	Complex rank	Number of actin contacted hot spots	Number of helical residues in region 9-18	Number of turn residues in region 9-18	RMSD of region 9-18	RMSD of ^{22}LKKV^{25}
219	788	549	6	0	6	13.3	31.6
369	136	583	7	5	0	9.5	8.7
125	795	753	6	0	8	5.3	35.9
255	959	805	7	0	7	9.0	34.8
**428**	**317**	**1547**	**8**	**0**	**6**	**4.0**	**16.2**
411	579	2098	7	0	2	7.1	31.3
189	443	2173	7	0	2	6.0	26.8
279	730	2778	8	0	6	4.3	13.5
77	545	3203	6	0	8	8.8	18.5
279	927	3412	6	0	6	4.6	11.8
407	336	3541	7	3	3	9.7	14.5
198	312	3689	8	3	3	6.0	21.4
91	492	3702	7	0	8	8.1	29.5
212	896	3807	7	4	3	10.8	20.4
145	243	6613	6	0	1	11.4	23.2
407	167	7023	8	3	3	10.2	18.3

Table S2. Most probable 1:1 actin-domain V encounter complexes in which domain V segment 9-18 is in contact with at least 6 over 9 actin hot-spot residues and favorably oriented so that residues ^{22}LKKV^{25} can reach their cognate site on actin. RMSD (Å) are calculated over Cα atoms relative to N-WASP conformation in crystallographic structure 2VCP [3].

Cluster ID	Pose ID	Complex rank	Number of actin contacted hot spots	Number of helical residues in region 37-46	Number of turn residues in region 37-46	RMSD of region 37-46	RMSD of ^{50}LKSV^{53}
455	213	45	6	0	0	6.7	22.1
333	161	279	7	4	1	7.9	11.3
105	111	330	7	0	7	10.7	27.4
284	659	730	9	6	0	5.7	17.4
26	408	810	6	0	3	4.8	29.7
140	51	1009	6	0	6	12.5	26.0
113	756	1168	6	0	4	7.9	32.9
67	567	1366	8	0	1	8.2	30.2
105	535	1648	6	0	7	10.8	27.3
135	834	1656	7	0	4	9.5	27.3
212	864	1819	8	0	9	10.2	37.7
138	867	2963	6	4	2	7.8	27.8
**234**	**441**	**3129**	**6**	**6**	**0**	**3.4**	**29.8**
230	513	3623	6	0	7	10.6	25.0
455	825	4325	7	0	0	5.6	23.9
284	405	4686	8	6	0	10.5	16.9
518	95	6231	9	0	1	11.9	21.5
310	558	6889	8	3	4	6.7	17.8

Table S3. Most probable 1:1 actin-domain V encounter complexes in which domain V segment 37-46 is in contact with at least 6 over 9 actin hot-spot residues and favorably oriented so that residues ^{50}LKSV^{53} can reach their cognate site on actin. RMSD (Å) are calculated over Cα atoms relative to N-WASP conformation in crystallographic structure 2VCP [3].

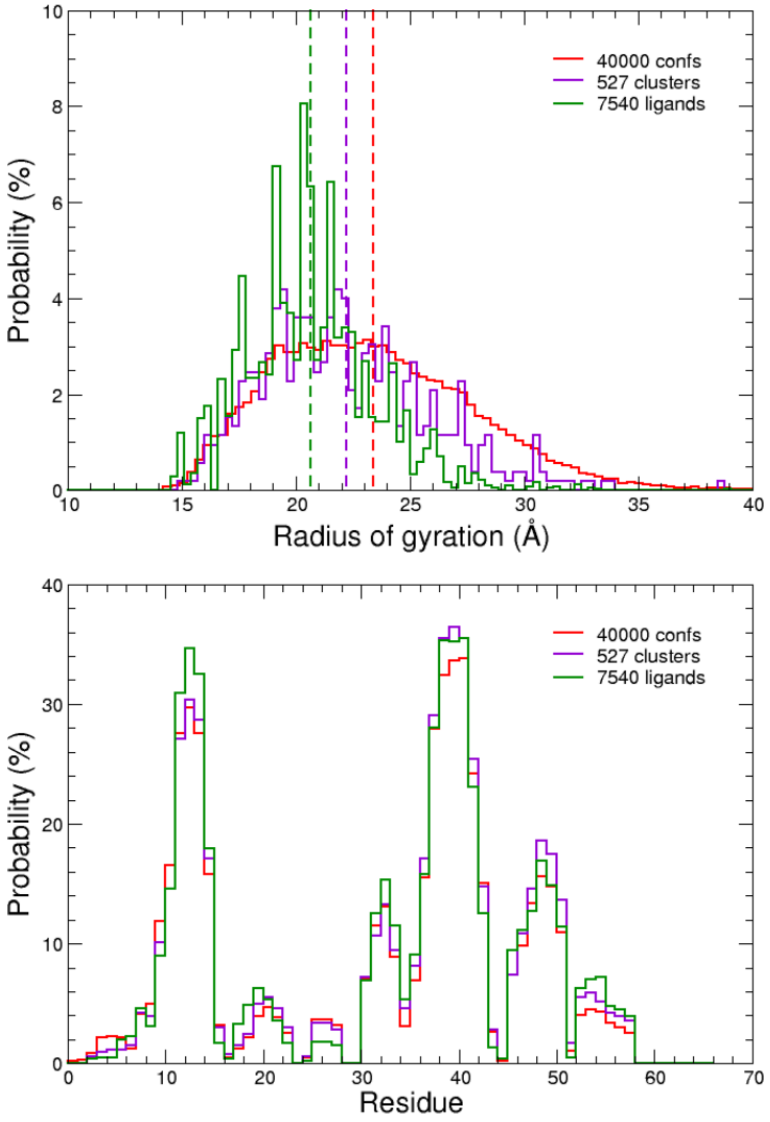

Figure S3. Comparison of N-WASP domain V residue probabilities to be in α-helix (bottom) and distributions of gyration (top) between the ensemble of initial 40 000 conformations (red), the 527 cluster representative structures (violet), and the 7 540 conformers in the most probable 2:1 actin-domain V complexes (green). Vertical dashed lines indicate average values of radius of gyration for the three conformational ensembles.

Cluster ID	Pose ID	Complex rank	Number of actin contacted hot spots	Number of helical residues in region 9-18	Number of turn residues in region 9-18	RMSD of region 9-18	RMSD of ^{22}LKKV^{25}
107	28	129	8	0	9	4.7	25.6
110	476	239	6	0	6	4.4	22.0
**370**	**34**	**255**	**6**	**6**	**3**	**4.2**	**26.0**
146	252	813	6	0	2	6.2	26.8
407	211	1116	6	3	3	9.5	14.8
132	249	1646	6	0	2	5.6	24.2
273	420	2199	6	0	6	7.4	25.4
411	1012	2780	6	0	2	6.8	30.1
145	978	3465	7	0	1	9.3	23.3
177	64	6419	7	0	2	5.9	36.8

Table S4. Most probable 2:1 actin-domain V encounter complexes in which segment 9-18 is in contact with at least 6 over 8 hot-spot residues of actin chain A and favorably oriented so that residues ^{22}LKKV^{25} can reach their cognate site on actin. RMSD (Å) are calculated over Cα atoms relative to N-WASP conformation in crystallographic structure 3M3N [4].

Cluster ID	Pose ID	Complex rank	Number of actin contacted hot spots	Number of helical residues in region 37-46	Number of turn residues in region 37-46	RMSD of region 37-46	RMSD of ^{50}LKSV^{53}
51	76	45	6	0	0	6.1	24.7
106	498	69	6	0	8	9.8	35.5
333	119	71	7	4	1	7.8	11.2
398	41	126	6	5	1	9.3	13.5
160	231	391	6	0	0	10.8	20.2
84	968	435	6	0	5	12.0	32.4
450	716	811	6	5	3	11.1	16.8
377	960	1440	6	0	1	7.4	25.2
227	811	2055	6	0	0	13.3	28.9
105	298	2349	6	0	7	6.4	36.2
89	75	2525	8	0	1	7.1	6.7
230	1006	3451	6	0	7	5.6	22.3
**131**	**403**	**3455**	**6**	**3**	**3**	**4.3**	**27.0**

Table S5. Most probable 2:1 actin-domain V encounter complexes in which segment 37-46 is in contact with at least 6 over 8 hot-spot residues of actin chain A and favorably oriented so that residues ^{50}LKSV^{53} can reach their cognate site on actin. RMSD (Å) are calculated over Cα atoms relative to N-WASP conformation in crystallographic structure 3M3N [4].

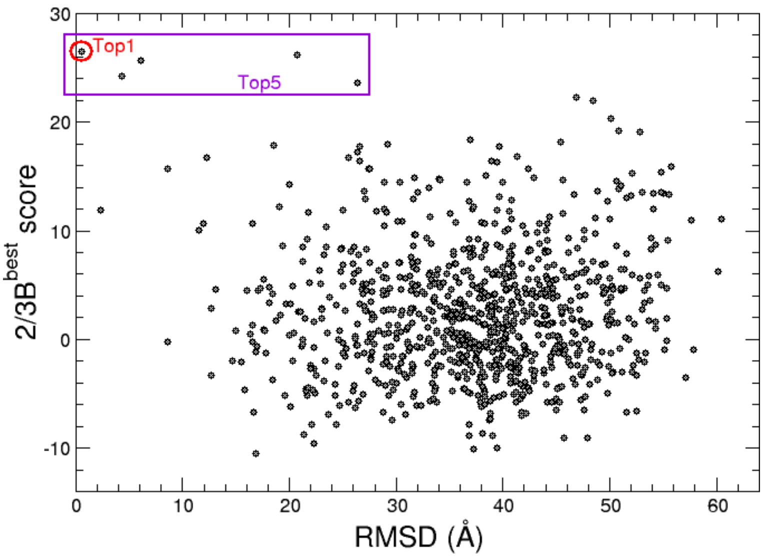

Figure S4. 2/3B^{best} score (best 2- and 3-body InterEvScore without inclusion of evolutionary information) of N-WASP segment 433-451 redocked into actin as a function of the ligand RMSD relative to the conformation found in X-ray structure 2VCP.

References

1. Chereau, D.; Kerff, F.; Graceffa, P.; Grabarek, Z.; Langsetmo, K.; Dominguez, R. Actin-bound structures of Wiskott–Aldrich syndrome protein (WASP)-homology domain 2 and the implications for filament assembly. *Proceedings of the National Academy of Sciences* **2005**, *102*, 16644–16649.
2. Lee, S.H.; Kerff, F.; Chereau, D.; Ferron, F.; Klug, A.; Dominguez, R. Structural basis for the actin-binding function of Missing-In-Metastasis. *Structure* **2007**, *15*, 145–155.
3. Gaucher, J.F.; Maugé, C.; Didry, D.; Guichard, B.; Renault, L.; Carlier, M.F. Interactions of Isolated C-terminal Fragments of Neural Wiskott-Aldrich Syndrome Protein (N-WASP) with Actin and Arp2/3 Complex. *Journal of Biological Chemistry* **2012**, *287*, 34646–34659.
4. Rebowski, G.; Namgoong, S.; Boczkowska, M.; Leavis, P.C.; Navaza, J.; Dominguez, R. Structure of a Longitudinal Actin Dimer Assembled by Tandem W Domains: Implications for Actin Filament Nucleation. *Journal of Molecular Biology* **2010**, *403*, 11–23.
5. Chen, X.; Ni, F.; Tian, X.; Kondrashkina, E.; Wang, Q.; Ma, J. Structural Basis of Actin Filament Nucleation by Tandem W Domains. *Cell Reports* **2013**, *3*, 1910–1920.
6. Scipion, C.P.M.; Ghoshdastider, U.; Ferrer, F.J.; Yuen, T.Y.; Wongsantichon, J.; Robinson, R.C. Structural evidence for the roles of divalent cations in actin polymerization and activation of ATP hydrolysis. *PNAS* **2018**, *115*, 10345–10350.
7. Ducka, A.M.; Joel, P.; Popowicz, G.M.; Trybus, K.M.; Schleicher, M.; Noegel, A.A.; Huber, R.; Holak, T.A.; Sitar, T. Structures of actin-bound Wiskott-Aldrich syndrome protein homology 2 (WH2) domains of Spire and the implication for filament nucleation. *Proc Natl Acad Sci USA* **2010**, *107*, 11757–11762.
8. Chen, C.K.; Sawaya, M.R.; Phillips, M.L.; Reisler, E.; Quinlan, M.E. Multiple Forms of Spire-Actin Complexes and their Functional Consequences. *J. Biol. Chem.* **2012**, *287*, 10684–10692.
